# A rare case report of recurrent primary intra-abdominal synovial sarcoma: An unusual manifestation

**DOI:** 10.1016/j.radcr.2023.10.039

**Published:** 2023-11-25

**Authors:** Hamd Zahra, Nosheen Kanwal, Muhammad Waleed Khalid, Anis ur Rehman, Khabab Abbasher Hussien Mohamed Ahmed, Muhammad Junaid Tahir

**Affiliations:** a Department of Radiology, Shaukat Khanum Memorial Cancer Hospital and Research Centre, Lahore, Pakistan; b Faculty of Medicine, University of Khartoum, Khartoum, Sudan

**Keywords:** Synovial sarcoma, Renal tumor, Malignancy, Imaging

## Abstract

Synovial sarcomas are a rare and aggressive subtype of soft tissue sarcomas that typically affects young adults and involves the extremities. Synovial sarcoma of the kidney is a rare and aggressive tumor with a poor prognosis, accounting for only 1% of all renal tumors. The imaging features of this tumor often overlap with those of other renal tumors, and a definitive diagnosis can only be made through immunohistochemical analysis. In this case report, we present the case of a 55-year-old female with left flank pain, who was diagnosed with primary renal synovial sarcoma following a left-sided radical nephrectomy. Despite initial successful surgical intervention, restaging scans showed local recurrence and metastatic disease, which was subsequently managed with 6 cycles of chemotherapy followed by radiation therapy with palliative intent. This case underscores the importance of early detection and aggressive management of rare renal tumors to improve patient outcomes.

## Introduction

Sarcomas are malignant tumors of mesenchymal origin and can be broadly categorized into soft tissue sarcomas and bone sarcomas [1]. Soft tissue sarcomas make up about 20% of pediatric and 1% of adult malignant tumors [2]. About 60% of these arise in limbs, 19% in the torso, 15% in the retroperitoneum, and 9% in the head and neck region [3]. Histologically they contain cells from various sites including adipose tissue, blood vessels, muscles, cartilage and bone. Abdominal soft tissue sarcomas are very rare and consist of about 0.1% of adult malignant tumors [2]. They can arise from the retroperitoneum, peritoneal cavity or abdominal wall. Among the renal sarcomas, the most common histological subtype is leiomyosarcoma, accounting for 40%-60% of the reported cases [4]. Primary renal synovial sarcoma is an extremely rare diagnosis and the first reported case in literature was described in 2000 [5]. The clinical presentation of renal synovial sarcoma is nonspecific and includes localized flank or back pain, hematuria, or palpable abdominal mass. Magnetic resonance imaging (MRI) is the imaging modality of choice generally the sarcomas, owing to the better visualization of the soft tissues. Synovial sarcoma is more common in young adults and has an overall poor prognosis. There is a high frequency of associated relapse and metastasis [6].

## Case presentation

A 55-year-old female presented with a history of left flank pain for three months, which worsened with time. There was no history of hematuria, significant weight loss, anorexia, cough, hemoptysis, or bone pain. On physical examination, there was no discrete palpable abdominal mass at the time of presentation.

**Fig. 1:**
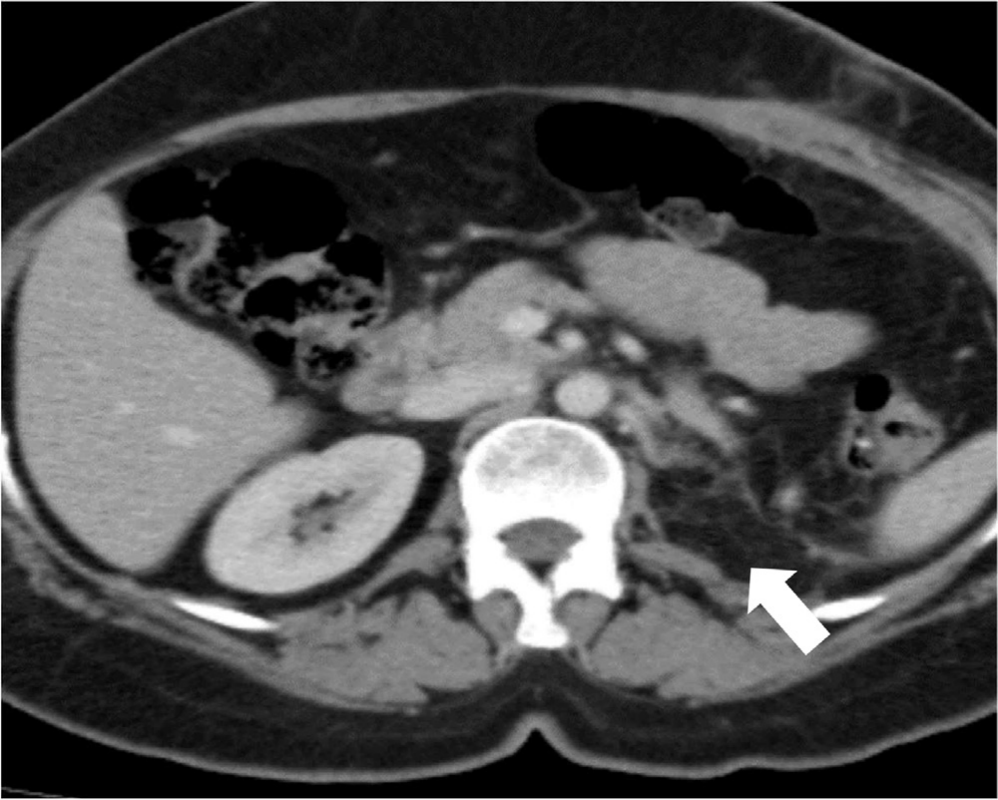
Postoperative CT scan axial image showing left nephrectomy with postsurgical changes (arrow) in left renal bed.

**Fig. 2:**
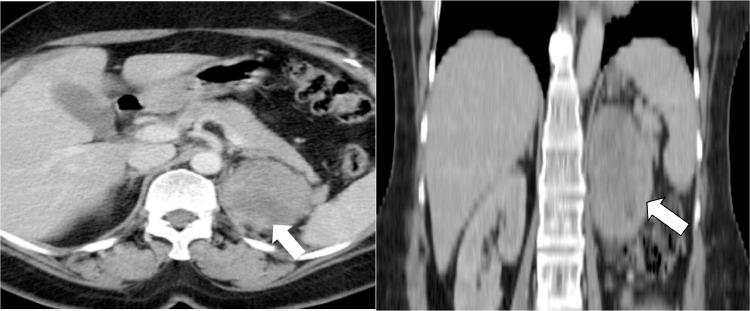
Three-month follow-up CT scan showing interval development of enhancing soft tissue mass (arrow, axial, and coronal view) in the left nephrectomy bed compatible with recurrent disease.

**Fig. 3:**
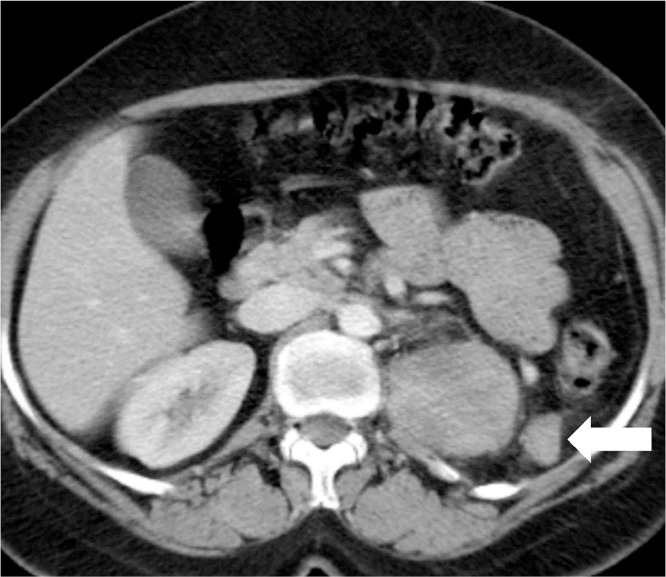
Three-month follow-up CT scan showing interval development of enhancing peritoneal deposit (white arrow) near soft tissue mass in the left nephrectomy bed.

**Fig. 4:**
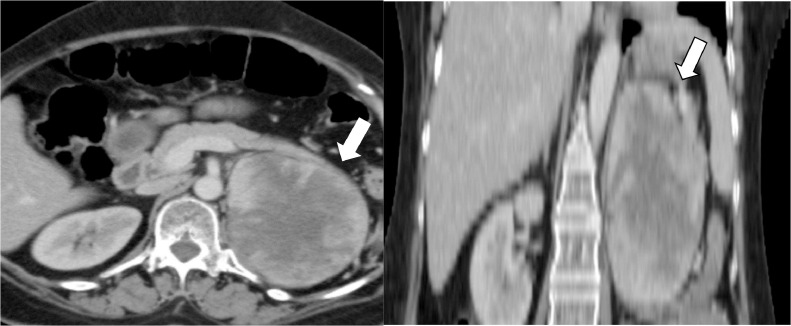
Five-month follow-up CT scan showing interval increase in the size of soft tissue mass (white arrow, axial, and coronal view) in the left nephrectomy bed.

Ultrasound abdomen was done for a workup which showed a left renal lower pole mass. Computed tomography (CT) abdomen was performed which showed a left renal mass without any extra renal extension or vascular involvement. The patient underwent a left radical nephrectomy. Histopathology showed a malignant neoplasm with a tumor invading the perirenal fat. The renal sinus fat, vascular and ureteric margins were free of tumor. Histopathology and immunocytochemistry showed it to be synovial sarcoma. The immediate postoperative CT scan showed postsurgical changes in the left nephrectomy bed without any evidence of metastatic disease (Fig. 1). However, a 3-month follow-up CT scan of the chest and abdomen showed a huge enhancing soft tissue mass measuring 7.9 × 8 × 8.6 cm in the left nephrectomy bed compatible with recurrent disease (Fig. 2). Further, small nodules were also noted adjacent to this lesion, with another large peritoneal mass suggestive of extensive peritoneal disease (Fig. 3). Five-month follow-up CT scan also showed an interval increase in the size of soft tissue mass measuring 8 × 9 × 11 cm in the left nephrectomy bed (Fig. 4). The patient was offered palliative chemotherapy in the form of 3 cycles of doxorubicin followed by 3 cycles of gemcitabine and docetaxel. The imaging showed progressive disease so further radiation therapy was offered to the patient was followed with palliative intent.

## Discussion

Synovial sarcomas are intermediate to high-grade malignant soft tissue tumors. They were so named initially because of their origin from the soft tissues around the knee joint. However, they can be present in any location, including the chest wall, abdomen, head, and neck. Most commonly they occur in the ages 20-*40s* and are 2.5%-10% of all soft tissue sarcomas [7]. Patients usually present with deep-seated slow-growing soft tissue masses. Most synovial sarcomas are associated with chromosomal translocation t(x;18) (p11.2; q11.2) producing SS18-SSX1, -SSX2 or -SSX4 fusion genes [8]. Histologically, they are divided into 4 subtypes: biphasic (20%-30%), monophasic fibrous (50%-60%), monophasic epithelial (rare) and poorly differentiated (15%-25%) [7]. Immunohistochemistry is useful in differentiating these tumors from most other sarcomas as they are positive for epithelial markers like keratins. Distant metastases (40%-70%) are frequently seen, common sites including lungs, bones, and regional lymph nodes [9]. However, in the case presented above, peritoneum was the only site of metastatic disease involvement.

The most common CT appearance is a heterogeneous, enhancing, deep-seated soft tissue mass with internal necrosis or hemorrhage. MRI is the ideal modality for the local staging of tumors. The mass can be heterogeneous or isointense to the muscles on T1 with variable enhancement patterns that can be diffuse, heterogeneous, or peripheral [10]. On T2, they are mostly hyperintense and can show internal fluid-fluid levels due to bleeding. Treatment is usually aggressive with surgery and adjuvant radiotherapy or chemotherapy [6]. Local recurrence is common. Tumors located within the trunk or head and neck are associated with poor prognosis. On the other hand, small size, younger age, presence of calcifications, and location in the extremities are factors associated with a good prognosis.

Primary renal sarcomas are rare neoplasms that account for 1% of all renal tumors [11]. The sarcomas that involve the kidney include rhabdomyosarcoma, malignant fibrous histiocytoma, fibrosarcoma, angiosarcoma, hemangiopericytoma, and rarely synovial sarcoma. The differential diagnosis of primary renal synovial sarcoma on histopathology includes sarcomatoid renal cell carcinoma and primary retroperitoneal soft tissue sarcomas with secondary renal involvement. Immunohistocytochemistry and genetic analysis help in establishing diagnosis. Primary renal synovial sarcoma occurs between the ages of 15 and 71, with equal incidence in both sexes [11]. This tumor has very aggressive behavior and a poor prognosis. The treatment protocol consists of adjuvant ifosfamide chemotherapy programs in conjunction with radical nephrectomy. Previously reported cases in the literature have shown good outcomes with these treatment regimens. Puj et al. [12] described a case of large renal synovial sarcoma treated with neoadjuvant ifosfamide and doxorubicin chemotherapy that resulted in the downstaging of the tumor and made surgical resection possible. Another case report by Chediak et al. [13] showed complete restumoron of a metastatic renal synovial sarcoma with surgery and doxorubicin and ifosfamide chemotherapy. Another approach is the use of preoperative renal artery embolization to reduce the tumor bulk as reported by Lohani et al. [14]. However, in most cases, treatment is only for palliative purposes due to the aggressive nature of the tumor.

One notable limitation of this case report is that prenephrectomy imaging data is unavailable as the patient presented to our institution following a nephrectomy performed at an outside hospital, and despite our diligent efforts, we were unable to obtain access to any previous imaging records. However, we have strived to compensate for this constraint by providing a thorough description of the postnephrectomy imaging findings and clinical history to offer insights into this rare case of primary renal synovial sarcoma.

## Conclusion

In conclusion, this case report highlights the importance of considering rare entities like synovial sarcoma in the differential diagnosis of renal masses, especially in patients without typical symptoms. The recurrence of disease in the left nephrectomy bed and extensive peritoneal involvement observed in the follow-up imaging underscores the aggressive nature of this tumor. The need for continued surveillance and a multidisciplinary approach to care is emphasized in such cases. It also highlights the need for early detection and aggressive treatment of synovial sarcoma to improve patient outcomes.

## Authors’ contributions

H.Z., A.U.R, and N.K conceived and designed the case report and were responsible for data collection and acquisition of data. H.Z, N.K, M.W.K, and M.J.T performed the literature review and wrote the manuscript. A.U.R, K.A.H and M.J.T reviewed and critically revised the manuscript. All authors have approved the final manuscript.

## Patient consent

This case report was conducted with the approval of the institutional review board (EX-28-10-22-02) and written informed consent for the publication of this case report was obtained from the patient. All patient data were de-identified to maintain confidentiality.
